# Hong Kong Women Project a Larger Body When Speaking to Attractive Men

**DOI:** 10.3389/fpsyg.2021.786507

**Published:** 2022-01-05

**Authors:** Albert Lee, Eva Ng

**Affiliations:** ^1^Department of Linguistics and Modern Language Studies, The Education University of Hong Kong, Tai Po, Hong Kong SAR, China; ^2^Department of Linguistics, The University of Hong Kong, Pokfulam, Hong Kong SAR, China

**Keywords:** body size projection, Cantonese, sexual selection, sociophonetics, vocal attractiveness

## Abstract

In this pilot study we investigated the vocal strategies of Cantonese women when addressing an attractive vs. unattractive male. We recruited 19 young female native speakers of Hong Kong Cantonese who completed an attractiveness rating task, followed by a speech production task where they were presented a subset of the same faces. By comparing the rating results and corresponding acoustic data of the facial stimuli, we found that when young Cantonese women spoke to an attractive male, they were less breathy, lower in fundamental frequency, and with denser formants, all of which are considered to project a larger body. Participants who were more satisfied with their own height used these vocal strategies more actively. These results are discussed in terms of the body size projection principle.

## Introduction

Having an attractive voice is useful because listeners tend to associate it with an attractive face (Hughes and Miller, 2016), a likeable personality (Zuckerman and Driver, 1989), and assign it higher health ratings (Albert et al., 2021). It has been reported that physical attractiveness leads to advantages in situations such as dating (Berscheid et al., 1971), job applications (Watkins and Johnston, 2000), promotion (Chung and Leung, 1988), elections (Jäckle et al., 2020), and is associated with more social support (Sarason et al., 1985). While one’s physical appearance cannot be easily altered at least in the short run, adjusting their own voice is an immediately possible alternative. Therefore, a good understanding of vocal attractiveness is of practical, social, and theoretical importance. So far, researchers have identified the characteristics of an attractive voice in perception experiments (e.g., by rating voice stimulus), but whether speakers choose to speak in the same preferred voice is an open question. This study approached this lesser-studied aspect of vocal attractiveness by studying how Cantonese women from Hong Kong choose their vocal strategies when addressing attractive vs. unattractive men.

### Averageness vs. Body Size Projection

Two seemingly competing hypotheses seek to account for the phonetics of an attractive voice, namely the averageness hypothesis and body size projection. The former stems from the ‘averaging attractiveness phenomenon’ and argues that voices similar to the population average are considered more attractive (see review in Belin, 2021). From an evolutionary point of view, the average voice may signal good genes as it has withstood evolution and adaptive changes to become the norm of the population, much like the average face appearing to signal high fitness (Langlois and Roggman, 1990; Thornhill and Gangestad, 1999). From a perceptual perspective, the average voice may be easier to process as it resembles a central voice prototype based on which voice identities are encoded, as is the case for face (Winkielman et al., 2006).

Meanwhile, the body size projection principle (Morton, 1977) contends that animals use their voice to project different body sizes to serve different communicative functions (e.g., small projected body to show appeasement, large to express hostility). Extending this principle, subsequently Xu et al. (2013) found that an attractive male voice to female English listeners was one that sounded large, vice versa for a female voice to male listeners. However, this does not mean that, for example, perceived attractiveness would monotonously increase with a smaller projected female body – extremely high fundamental frequency (*f*_o_ henceforth, i.e., the acoustic correlate of pitch) was not judged as the most attractive in Xu et al. (2013), possibly because the very small projected body started to sound more child-like than attractive. All in all, it appears that an attractive voice is one that resembles the population average, with specific projected body sizes (larger for male speakers, smaller for female speakers) adding enhancing effects, provided they do not deviate too much from the average.

### Acoustic Correlates of Body Size

In general, there is an inverse relationship between body size and *f*_o_ (Morton, 1977) as well as formant dispersion (Fitch, 1997). *f*_o_ is the frequency at which membranes (the vocal folds in the case of humans) vibrate (see Lee and Mok, 2021 for a recent review), and is determined by body size – “(t)he larger the animal, the lower the sound frequency it can produce” (Morton, 1977, p. 864). Formant dispersion, or the averaged difference between successive formant frequencies, reflects one’s vocal tract length (Fitch, 1997), and in turn body size. The shorter the vocal tract, the further apart the speaker’s formants. As for *f*_o_ *range*, the use of a larger *f*_o_ range is associated with both cooperativeness (*cf*. “the effort code,” Gussenhoven, 2016) and happiness (Xu et al., 2013), in turn likely a smaller body which signals less threat. In terms of voice quality, breathy voice (acoustically manifested in spectral parameters such as “H1–A1” and “H1–A3”) is argued to be acoustically more similar to pure tone compared with voice qualities such as modal voice (Xu et al., 2013), and signals a small body according to Morton (1977). Conversely, creaky voice (main acoustic correlates: “jitter” and “shimmer”) is typically argued to be associated with masculinity and authority (see Yuasa, 2010 for a review).

### Cross-Linguistic Variation in Preferences in Voices

The acoustic correlates of an attractive voice have been extensively studied in recent years. To male English listeners, an attractive female voice is high in *f*_o_, breathy, and with wide formant dispersion, all of which signal a small body; to female English listeners, an attractive male voice (i) is low in *f*_o_ and (ii) has narrow formant dispersion, both signaling a large body, but (iii) is also breathy, signaling a smaller body (Xu et al., 2013), presumably to neutralize some of the hostility accompanying the large projected body.

It has been reported that the creaky voice is increasingly used by American female speakers in recent years (Yuasa, 2010). Although this seems to deviate from the body-size projection principle, as creakiness is considered to be associated with a large body, there is also evidence that the use of creaky voice by American women is considered less attractive than a normal speaking voice (Anderson et al., 2014). Therefore, it seems to suggest instead that speakers’ vocal strategies do not necessarily have to align with what the opposite sex considers attractive.

Apart from Xu et al. (2013), comparable perception studies on non-Western populations include Japanese (Xu et al., 2017) and Mandarin (Xu and Lee, 2018), which demonstrated cross-linguistic variations in the acoustic cues to an attractive voice. These studies found that while the general principles of body size projection in accounting for patterns in voice preferences appeared to hold, there were also language-specific deviations. For example, in Mandarin and Japanese, a narrow *f*_o_ range (which signals a larger body) was found to be unattractive to both male and female listeners alike.

Although the perception of vocal attractiveness in western societies is relatively well understood, there is much less production data available, let alone from non-Western populations. To the best of our knowledge, to date there is no production study on vocal attractiveness in Cantonese. This study serves to fill this gap. While perception studies are useful for identifying the effect of individual acoustic cues, production data are essential as they show how these cues interact in everyday speech. In addition, production data can shed light on individual variability, which is increasingly important with the emergence of statistical tools capturing speakers as a random factor.

### Hypotheses

Based on the studies reviewed above, we expected that female Cantonese speakers would use vocal strategies to signal a small body (Hypotheses 3 and 4) when addressing an attractive male, but they might also be creaky (related to Hypotheses 1, 2, 5, and 6) like their American counterparts. The seemingly arbitrary prediction of creakiness is based on two reasons: (i) we tested well-educated young women in Hong Kong where the influence of western (including American) culture is prevalent (Louie, 2010), and (ii) the effect of voice quality in neutralizing one’s projected body size was also observed in female English listeners’ preferences in a male voice (Xu et al., 2013). Therefore, in this study we tested the following hypotheses (see Table 1):

**TABLE 1:** *Working hypotheses* (prediction for the attractive facial stimulus condition).

	Perceptual property	Acoustic correlate	Hypothesis	Projected body
Hypothesis 1	Breathiness	H1-A1	Decrease	Large
Hypothesis 2		H1-A3	Decrease	Large
Hypothesis 3	Apparent vocal tract length	Formant disp.	Increase	Small
Hypothesis 4	Pitch	Median *f*_o_	Increase	Small
Hypothesis 5	Creakiness	Jitter	Increase	Large
Hypothesis 6		Shimmer	Increase	Large

Hypothesis 1 and 2 are related to the use of breathy voice. As the decrease in energy at higher frequencies from the first harmonic (or H1) is the greatest for breathy voice and the least for creaky voice (see review in Gordon and Ladefoged, 2001), we expected to see decreased H1–A1 (where A1 stands for amplitude of the first formant) and H1–A3 in the attractive face condition (i.e., less breathy as we are also hypothesizing increased creakiness in Hypotheses 5 and 6). Here we included multiple spectral parameters (i.e., both H1–A1 and H1–A3) to ensure reliability of our results (*cf*. Kreiman et al., 2007). Formant dispersion (Hypothesis 3) is inversely related to vocal tract length, thus in the attractive face condition we expect to see more dispersed formants that project a shorter vocal tract, in turn a smaller body. Hypothesis 4 is based on the assumption that Cantonese women would project a smaller body with higher median *f*_o_ when addressing an attractive man. Finally, while there are different types of creaky voice (Redi and Shattuck-Hufnagel, 2001), each with its own acoustic properties, as working hypotheses (Hypothesis 5 and 6) we hypothesized that Cantonese women would exhibit more cycle-to-cycle variability in the attractive face condition, thus increased jitter and shimmer (i.e., more creakiness).

## Methods

### Participants

Nineteen women participated in this study. They were all recruited in Hong Kong, speaking Cantonese as their first language, and university-educated (either then-current students or recently graduated). They aged between 19 and 25, and self-declared as heterosexual. All of them also spoke English and Mandarin as second languages. Their mean height was 159.4 cm (SD ± 4.4). Participation was voluntary and no one received any monetary remuneration. No one reported any (history of) speech and hearing impairment.

### Warm-Up Task

This study comprised three tasks: warm-up, facial attractiveness rating, and speech production task. All tasks were completed in the same session in a quiet room on university campus. Participants were recorded using a Logitech H340 microphone at a sampling rate of 44.1 kHz.

During the warm-up session, participants were asked to say the semantically neutral utterance 你好啊，你讀咩科嫁？ “Hello. What is your major?” three times without being presented any visual stimuli. The purpose of this task was to familiarize the participants with main production task, which will be described below.

### Facial Attractiveness Rating

Fifty different male facial stimuli were used. We only included faces of East Asian ethnicities as their features are more familiar to our participants (*cf*. Coetzee et al., 2014). Forty of the faces were relatively attractive Asian male faces (celebrities and otherwise). The remaining stimuli were relatively less attractive male faces (again including celebrities). The images of male celebrities were those from Hong Kong, Korea, Japan, and Mainland China. All stimuli were publicly available images obtained from the Internet.

Participants were asked to rate the attractiveness of these 50 faces on a 1 ∼ 10 scale (10 = most attractive) and write down their response on an answer sheet. They were told to base their ratings purely on how much they were attracted to each face, and to ignore any past knowledge of the respective males or experience they might have with people of similar appearances. Stimuli were presented in a randomized order in Microsoft Powerpoint slides.

### Production Task

Based on the ratings from above, for each participant the five most attractive and five least attractive faces were used as target stimuli in a subsequent production task. In the event of faces with the same rating, those that were presented later were chosen. Each face was presented three times on separate occasions in random order. Participants were instructed to imagine themselves in a classroom setting, and that the male face was of a classmate sitting next to them. Participants were then to ask the male classmate 你好啊，你讀咩科嫁？ “Hello. What is your major?” From each participant, 30 utterances were recorded. Recordings were subsequently analyzed using ProsodyPro (Xu, 2013 ver. 5.7.2), which allows manual checking of vocal pulses and automatically extracts numerous acoustic measurements, as will be presented below.

### *Post hoc* Questionnaire

Preliminary data analysis revealed a bimodal distribution which was seemingly related to participants’ height. Specifically, we seemed to observe that taller participants seemed to behave in the opposite direction from the rest. To verify this, we sent out a questionnaire to gather information on participants’ height and how satisfied they were about it. There were four questions in the questionnaire: (1) “How tall are you?,” (2) “On a scale of 1 to 10, how satisfied are you about your own height?,” (3) “If you are not satisfied, how much taller/shorter would you like to be (in centimeters)?,” and (4) “What are you doing to address your unsatisfactory height (e.g., wearing high heels)?” All participants bar one responded (i.e., *N* = 18). Based on their response, participants were then classified in terms of how satisfied they were about their height, namely (H)ighly satisfied, (M)oderately satisfied, and (L)east satisfied. There were six participants in each category. The correlation between participants’ height and their satisfaction with their own height was nearly but not significant, *r*_*s*_ = 0.446, *N* = 18, *p* = 0.063.

## Results

**FIGURE 1:**
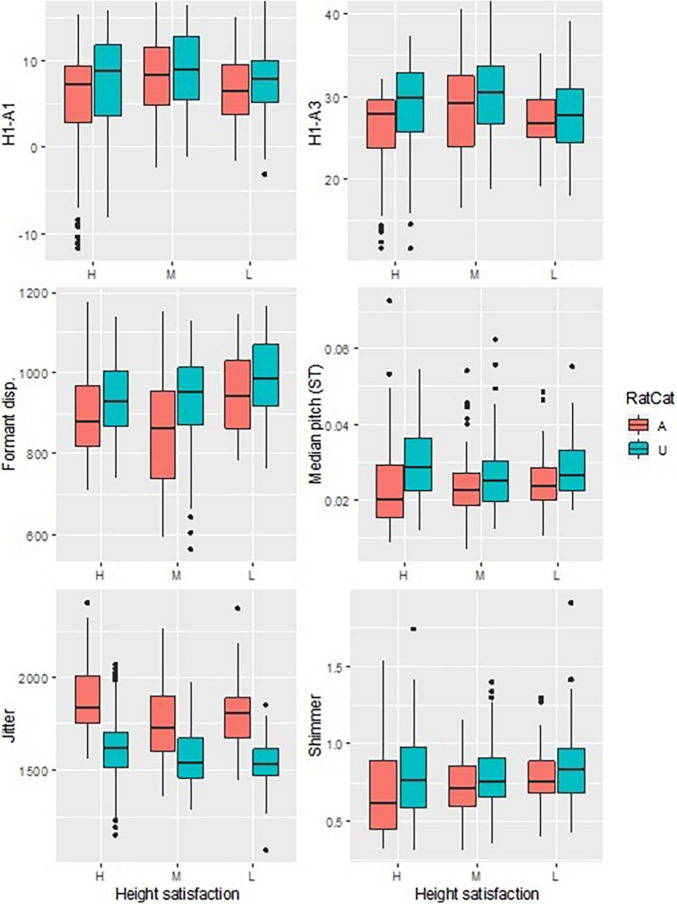
Boxplots comparing acoustic correlates of projected voices in attractive (A) vs. unattractive (U) facial stimulus conditions. H, M, L stand for highly, moderately, least satisfied with speakers’ own height.

We set out to test six hypotheses (see Table 1) to examine whether Cantonese women project a small body when addressing an attractive male. Results are shown in Figure 1, where attractive (A) and unattractive (U) facial stimuli are compared (coral and turquoise, respectively) for each acoustic correlate of vocal attractiveness. The *X*-axis of Figure 1 represents how much speakers were satisfied with their own body height (converted into the three categories H, M, L, with H being the most satisfied). See also Supplementary Figure 1 for corresponding boxplots with height satisfaction contrasts collapsed.

For voice quality, H1–A1 was higher for unattractive stimuli, indicating more use of breathiness when participants spoke to an unattractive face; the same was true for H1–A3. Cantonese women also appeared to lengthen their vocal tract with denser formants in the Attractive condition, thus projecting a larger body. Similarly, participants’ median *f*_o_ was lower in the Attractive condition. In terms of creakiness, participants showed higher jitter but lower shimmer in the Attractive condition.

Initial exploratory data analysis (based on visual inspection of Supplementary Figure 2) revealed substantial individual variability in vocal strategies. Therefore, for each acoustical parameter in Figure 1, we fitted a linear mixed effects model using the *lmerTest* package in R (Kuznetsova et al., 2017, ver. 3.1-3) to model by-speaker variations. Model summaries are shown in Table 2. All models contained the continuous predictor of Attractiveness (rating of male facial stimuli). In some models, we also included the interaction between Attractiveness and desired change in height (see Question 3 in §2.5), which appeared to be a good heuristic of the individual variation. No other manipulation of the data was performed. All models included by-speaker random intercepts; most also included by-speaker random slope for Attractiveness (except for shimmer, in which model including the random slope for Attractiveness would lead to non-convergence).

**TABLE 2:** Model summaries for different acoustic correlates.

		Fixed effects					Random effect
							SD
		*Est.*	*SE*	*df*	*t*	*p*	Speaker
H1–A1	(Intercept)	8.218	0.930	18.056	8.840	**<0.001**	3.944
	Attract.	−0.417	0.119	17.124	−3.499	**0.003**	0.317
	Attract.:DesChg	0.055	0.021	17.542	2.578	**0.019**	
H1–A3	(Intercept)	29.119	1.218	17.946	23.904	**<0.001**	5.218
	Attract.	–0.444	0.135	19.920	−3.277	**0.004**	0.403
	Attract.: DesChg	0.055	0.022	17.519	2.453	**0.025**	
F1–F3	(Intercept)	968.015	21.398	17.765	45.238	**<0.001**	89.719
	Attract.	−8.909	2.179	15.555	−4.088	**0.001**	8.377
Median *f*_o_	(Intercept)	0.029	0.001	18.489	22.055	**<0.001**	0.005
	Attract.	–0.001	<0.001	15.992	−4.265	**0.001**	0.001
Jitter	(Intercept)	1527.525	36.888	17.853	41.410	**<0.001**	156.64
	Attract.	44.184	5.347	17.882	8.263	**<0.001**	15.78
	Attract.:DesChg	−1.970	0.903	16.526	−2.180	**0.044**	
Shimm.	(Intercept)	0.823	0.036	22.020	23.012	**<0.001**	0.144
	Attract.	–0.015	0.003	542.600	−4.692	**<0.001**	
	Attract.: DesChg	0.001	0.001	480.400	2.011	**0.045**	

*“Attract.” stands for facial attractiveness rating (1–10, 10 = the most attractive). “DesChg” stands for desired change in height (in cm). Significant fixed effects are in bold.*

Table 2 shows that there was a significant main effect of Attractiveness (*p* < 0.005 for all cases) on all acoustical correlates of vocal attractiveness analyzed. This indicates that, after taking into account by-speaker variation, in general an attractive male face elicited significantly less breathiness (lower H1–A1 and H1–A3), longer vocal tract (denser formant dispersion), lower median *f*_o_, less regular cycle-to-cycle variation in *f*_o_ (higher jitter) but more regular cycle-to-cycle variation in amplitude (lower shimmer). In addition, there was a significant interaction between Attractiveness and desired change in height in all voice quality-related measurements. It can be seen in Figure 1 and Table 3 that the contrast between attractive and unattractive faces in terms of acoustic correlates were bigger for speakers who were satisfied with their own height (in bold in Table 3). The opposite parameter estimates of the main effect of Attractiveness and the interaction term in Table 2 may be understood from the fact that those desiring the largest change in height are the least satisfied, and are using vocal strategies *less* differently to address attractive vs. unattractive males (*cf*. Table 3).

**TABLE 3:** Summary of differences (attractive less unattractive facial stimuli) in each acoustic correlate by speakers’ satisfaction in their own height (H being the most satisfied).

	Attractive *less* unattractive facial stimuli			
	H1–A1	H1–A3	Jitter	Shimmer
H	**−2.308**	**−2.627**	**27.447**	**−0.025**
M	−0.781	−1.773	18.349	−0.020
L	−0.787	−0.253	24.565	−0.016

*Significant fixed effects are in bold.*

## Discussion

### Summary of Findings

This study explored how Cantonese women projected their voice when speaking to an attractive vs. unattractive face. Results showed that, in the attractive face condition, most acoustic cues pointed to a larger body (except Hypothesis 6). Participants were significantly less breathy (lower H1–A1 and H1–A3) in the attractive condition, supporting Hypothesis 1 and 2. In terms of vocal tract length, participants showed narrower formant dispersion in the attractive condition, signaling a larger body, rejecting Hypothesis 3. Their median *f*_o_ was also significantly lower when addressing an attractive face, thus rejecting Hypothesis 4. For creaky phonation, in the attractive face condition there was greater jitter but smaller shimmer, thus supporting Hypothesis 5 but not Hypothesis 6. For all voice quality-related measurements (i.e., analyses of breathiness and creakiness), there was a significant interaction between facial attractiveness rating and desired change in height.

### Body Size Projection

As reported in Xu et al. (2013), male English listeners judged small-sounding acoustic cues to be more attractive, so even with cross-linguistic variation one would have expected Cantonese women to at least use some small-sounding cues in their production. Rather unexpectedly, in our data participants seemed to be always trying to project a large-sounding voice instead when speaking to an attractive face, unlike what the body size projection account would have predicted. This is reminiscent of the prevalent use of creaky voice by female American speakers, despite that creakiness is considered unattractive (Anderson et al., 2014). The case of creaky voice in American female speech shows that speakers do not necessarily use vocal strategies that listeners typically consider attractive – knowingly or otherwise. Another conceivable speculation is that speakers were taking into account social factors (classroom setting with friends nearby, interlocutor being a classmate), such that they deliberately avoided sounding too eager in front of an attractive potential mate. This speculation, needless to say, needs to be carefully verified.

In our initial analysis, we had the impression that speakers’ height might affect their vocal strategies – this was confirmed in Table 3. For all voice quality-related acoustic cues, participants who were satisfied with their own height manifested a larger contrast between the attractive and the unattractive stimulus conditions. Our data thus seem to suggest that although female Cantonese speakers have the same set of vocal strategies for attractive vs. unattractive mates, it is those who are confident in their own height that are using them more actively.

### Caveats

Participants in this study were well-educated young women who had been exposed to western culture since a very young age. They also spoke fluent English and Mandarin as second languages. This group of speakers thus represents only a subset of the local population. It is also noteworthy that when they took part in the production task, they had already been primed to think about attractiveness during the rating task – this could possibly have affected how they spoke. Finally, as is clear from Supplementary Figure 2, there is substantial individual variability in terms of vocal strategies. Therefore, this study may benefit from a larger sample than 19 speakers.

### Suggestions for Future Research

Future studies should look at other groups of speakers in the community, such as older monolingual speakers. Another potentially interesting factor to investigate would be the effect of menstrual cycle on speech production. To the best of our knowledge, to date there is only preliminary data on how the menstrual cycle affects voice quality in Cantonese women (Li, 2016). Understanding how physiological factors interact with vocal attractiveness would shed new light on this issue. Finally, it would also be useful to verify the present findings with articulatory data, such as electroglottography (i.e., laryngograph).

## Conclusion

This pilot study has found that young Cantonese women projected a large-sounding voice when speaking to an attractive male face. This seems to disagree with the widely held body size projection principle which states that an attractive female voice is small-sounding. We also found that women who were confident in their own height adjust their voice more actively depending on the attractiveness of their mates. Further investigation is needed to understand the relationship between the present findings and those observed in other languages.

## Data Availability Statement

The raw data supporting the conclusions of this article will be made available by the authors, without undue reservation.

## Ethics Statement

The studies involving human participants were reviewed and approved by School of Humanities, the University of Hong Kong. The patients/participants provided their written informed consent to participate in this study.

## Author Contributions

EN conceived of the study supervised by AL. EN collected the data. AL analyzed the data and wrote the final manuscript. Both authors contributed to the article and approved the submitted version.

## Conflict of Interest

The authors declare that the research was conducted in the absence of any commercial or financial relationships that could be construed as a potential conflict of interest.

## Publisher’s Note

All claims expressed in this article are solely those of the authors and do not necessarily represent those of their affiliated organizations, or those of the publisher, the editors and the reviewers. Any product that may be evaluated in this article, or claim that may be made by its manufacturer, is not guaranteed or endorsed by the publisher.

## References

[B1] Albert G. Arnocky S. Puts D. A. Hodges-Simeon C. R. (2021). Can listeners assess men’s self-reported health from their voice? *Evol. Hum. Behav.* 42 91–103. 10.3109/09638288.2013.793750 23688293

[B2] Anderson R. C. Klofstad C. A. Mayew W. J. Venkatachalam M. (2014). Vocal fry may undermine the success of young women in the labor market. *PLoS One* 9:e97506. 10.1371/journal.pone.0097506 24870387 PMC4037169

[B3] Belin P. (2021). “On voice averaging and attractiveness,” in *Voice Attractiveness: Studies on Sexy, Likable, and Charismatic Speakers*, eds Weiss B. Trouvain J. Barkat-Defradas M. Ohala J. J. (Berlin: Springer), 139–149.

[B4] Berscheid E. Dion K. Walster E. Walster G. W. (1971). Physical attractiveness and dating choice: a test of the Matching Hypothesis. *J. Exp. Soc. Psychol.* 7 173–189. 10.1016/0022-1031(71)90065-5

[B5] Chung P.-P. Leung K. (1988). Effects of performance information and physical attractiveness on managerial decisions about promotion. *J. Soc. Psychol.* 128 791–801. 10.1080/00224545.1988.9924557

[B6] Coetzee V. Greeff J. M. Stephen I. D. Perrett D. I. (2014). Cross-cultural agreement in facial attractiveness preferences: the role of ethnicity and gender. *PLoS One* 9:e99629. 10.1371/journal.pone.0099629 24988325 PMC4079334

[B7] Fitch W. T. S. (1997). Vocal tract length and formant frequency dispersion correlate with body size in rhesus macaques. *J. Acoustical Soc. Am.* 102 1213–1222. 10.1121/1.421048 9265764

[B8] Gordon M. K. Ladefoged P. N. (2001). Phonation types: a cross-linguistic overview. *J. Phonet.* 29 383–406. 10.1006/jpho.2001.0147

[B9] Gussenhoven C. H. M. (2016). Foundations of intonational meaning: anatomical and physiological factors. *Top. Cogn. Sci.* 8 425–434. 10.1111/tops.12197 27016315

[B10] Hughes S. M. Miller N. E. (2016). What sounds beautiful looks beautiful stereotype: the matching of attractiveness of voices and faces. *J. Soc. Pers. Relationships* 33 984–996. 10.1177/0265407515612445

[B11] Jäckle S. Metz T. Wenzelburger G. König P. D. (2020). A catwalk to congress? appearance-based effects in the elections to the U.S. house of representatives 2016. *Am. Politics Res.* 48 427–441.

[B12] Kreiman J. E. Gerratt B. R. Antoñanzas Barroso N. S. (2007). Measures of the glottal source spectrum. *J. Speech Lang. Hear Res.* 50 595–610. 10.1044/1092-4388(2007/042) 17538103

[B13] Kuznetsova A. Brockhoff P. B. Christensen R. H. B. (2017). lmerTest package: tests in linear mixed effects models. *J. Statist. Software* 82 1–26.

[B14] Langlois J. H. Roggman L. A. (1990). Attractive faces are only average. *Psychol. Sci.* 1 115–121.

[B15] Lee A. Mok P. K. P. (2021). “Lexical tone,” in *The Cambridge Handbook of Phonetics*, eds Knight R.-A. Setter J. (Cambridge: Cambridge University Press), 185–208.

[B16] Lee A. Ng E. (2019). “Vocal attractiveness in cantonese: a production study,” in *Proceedings of the 19th International Congress of Phonetic Sciences (ICPhS 2019).* (Canberra, Act: Australasian Speech Science and Technology Association Inc).

[B17] Li C. H. (2016). Voice onset time in Cantonese women across the menstrual cycle. *J. Acoustical Soc. Am.* 140:3112.

[B18] Louie K. H. (2010). “Hong Kong on the move: creating global cultures,” in *Hong Kong Culture: Word and Image*, ed. Louie K. H. (Hong Kong: Hong Kong University Press).

[B19] Morton E. S. (1977). On the occurrence and significance of motivation-structural rules in some bird and mammal sounds. *Am. Nat.* 111 855–869. 10.1016/j.beproc.2009.04.008 19520235

[B20] Redi L. Shattuck-Hufnagel S. R. (2001). Variation in the realization of glottalization in normal speakers. *J. Phonet.* 29 407–429. 10.1080/02699200802394856 19148810

[B21] Sarason B. R. Sarason I. G. Hacker T. A. Basham R. B. (1985). Concomitants of social support?: social skills, physical attractiveness, and gender. *J. Pers. Soc. Psychol.* 49 469–480. 10.1037/0022-3514.49.2.469

[B22] Thornhill R. Gangestad S. W. (1999). Facial attractiveness. *Trends Cogn. Sci.* 3 452–460. 10562724 10.1016/s1364-6613(99)01403-5

[B23] Watkins L. M. Johnston L. (2000). Screening job applicants: the impact of physical attractiveness and application quality. *Int. J. Select. Assess.* 8 76–84. 10.1111/1468-2389.00135

[B24] Winkielman P. Halberstadt J. Fazendeiro T. Catty S. (2006). Prototypes are attractive because they are easy on the mind. *Psychol. Sci.* 17 799–806. 10.1111/j.1467-9280.2006.01785.x 16984298

[B25] Xu A. Lee A. (2018). Perception of vocal attractiveness by native Mandarin listeners. *Speech Prosody* 2018 344–348.

[B26] Xu A. Leung S.-S. Lee A. (2017). Universal vs. language-specific aspects in human vocal attractiveness: an investigation towards Japanese native listeners’ perceptual pattern. *Proc. Meet. Acoustics* 29 1–8.

[B27] Xu Y. (2013). “ProsodyPro: a tool for large-scale systematic prosody analysis,” in *Proceedings of Tools and Resources for the Analysis of Speech Prosody (TRASP 2013).* (Aix-en-Provence).

[B28] Xu Y. Lee A. Wu W.-L. Liu X. Birkholz P. (2013). Human vocal attractiveness as signaled by body size projection. *PLoS One* 8:e62397. 10.1371/journal.pone.0062397 23638065 PMC3634748

[B29] Yuasa I. P. (2010). Creaky voice: a new feminine voice quality for young urban-oriented upwardly mobile American women? *Am. Speech* 85 315–337. 10.1215/00031283-2010-018

[B30] Zuckerman M. Driver R. E. (1989). What sounds beautiful is good: the vocal attractiveness stereotype. *J. Nonverb. Behav.* 13 67–82. 10.1016/j.cub.2009.11.034 20129047

