# Supplementary material for: Hong Kong Women Project a Larger Body When Speaking to Attractive Men
Source: Front Psychol. 2022 Jan 5;12:786507. doi: 10.3389/fpsyg.2021.786507 (PMC8767052; doi:10.3389/fpsyg.2021.786507)
Supplement: Supplementary file 1 [file Image_1.pdf]

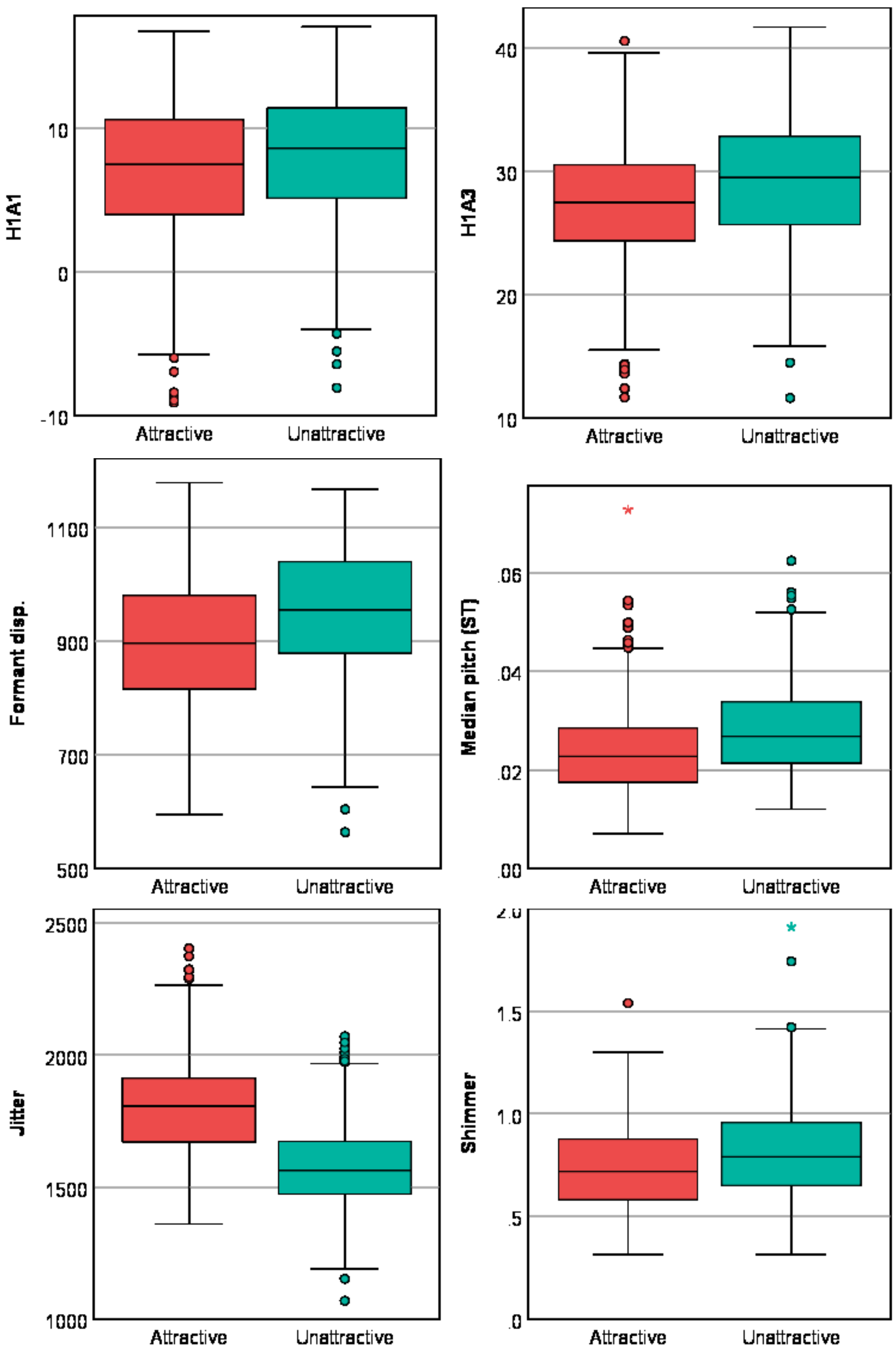

Supplementary Figure 1. Boxplots comparing acoustic correlates of projected voices in attractive vs. unattractive facial stimulus conditions with height satisfaction contrasts collapsed.
