# Supplementary material for: Hong Kong Women Project a Larger Body When Speaking to Attractive Men
Source: Front Psychol. 2022 Jan 5;12:786507. doi: 10.3389/fpsyg.2021.786507 (PMC8767052; doi:10.3389/fpsyg.2021.786507)
Supplement: Supplementary file 2 [file Image_2.pdf]

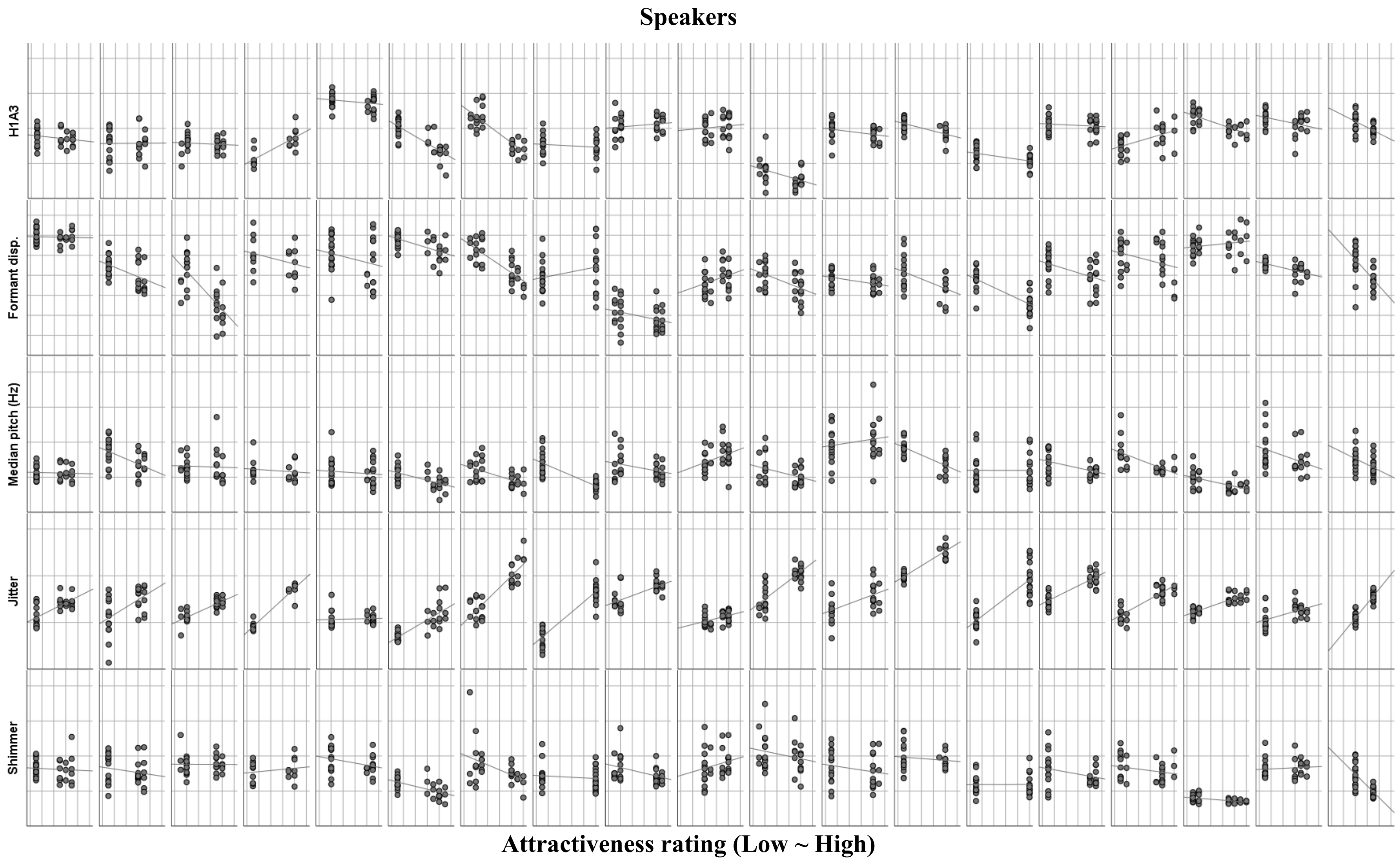

Supplementary Figure 2. Scatterplots showing the relationship between attractiveness ratings of facial stimuli and selected acoustic correlates of corresponding vocal productions.
